# Forest Loss is Accelerating Along the US Gulf Coast

**DOI:** 10.1007/s12237-021-01000-6

**Published:** 2021-09-09

**Authors:** Matthew J. McCarthy, Benjamin Dimmitt, Sebastian DiGeronimo, Frank E. Muller-Karger

**Affiliations:** 1 College of Marine Science, University of South Florida, 140 7th Avenue South, Saint Petersburg, FL 33701 USA; 2 Benjamin Dimmitt Photography, 22 DJ Drive, Fairview, NC 28730 USA

**Keywords:** Sea-level rise, Climate change, Ghost forest, Florida, Landsat

## Abstract

Sea-level rise is impacting the longest undeveloped stretch of coastline in the contiguous United States: The Florida Big Bend. Due to its low elevation and a higher-than-global-average local rate of sea-level rise, the region is losing coastal forest to encroaching marsh at an unprecedented rate. Previous research found a rate of forest-to-marsh conversion of up to 1.2 km^2^ year^−1^ during the nineteenth and twentieth centuries, but these studies evaluated small-scale changes, suffered from data gaps, or are substantially outdated. We replicated and updated these studies with Landsat satellite imagery covering the entire Big Bend region from 2003 to 2016 and corroborated results with in situ landscape photography and high-resolution aerial imagery. Our analysis of satellite and aerial images from 2003 to 2016 indicates a rate of approximately 10 km^2^ year^−1^ representing an increase of over 800%. Areas previously found to be unaffected by the decline are now in rapid retreat.

## Introduction

Coastal habitats are among the most vulnerable to climate disruption through sea-level rise, storm surge, and subsequent saltwater intrusion (Williams et al. 1999; Desantis et al. 2007; Chen et al. 2017; Kirwan and Gedan 2019). Recent observed rates of global warming of the atmosphere and ocean waters and subsequent sea-level rise find that observations agree with business-as-usual expectations (i.e., Intergovernmental Panel on Climate Change Assessment Report 5 RCP8.5), predict further global average acceleration, and note that local rates may differ substantially from the global average (IPCC 2014; Brown and Caldeira 2017; Sweet et al. 2017). As sea level rises, low-terrain tidal marshland migrates landward to encroach on and ultimately replace coastal forest, thereby creating “ghost forests” (Donnelly and Bertness 2001; Doyle et al. 2010; Koch et al. 2015).

**Fig. 1:**
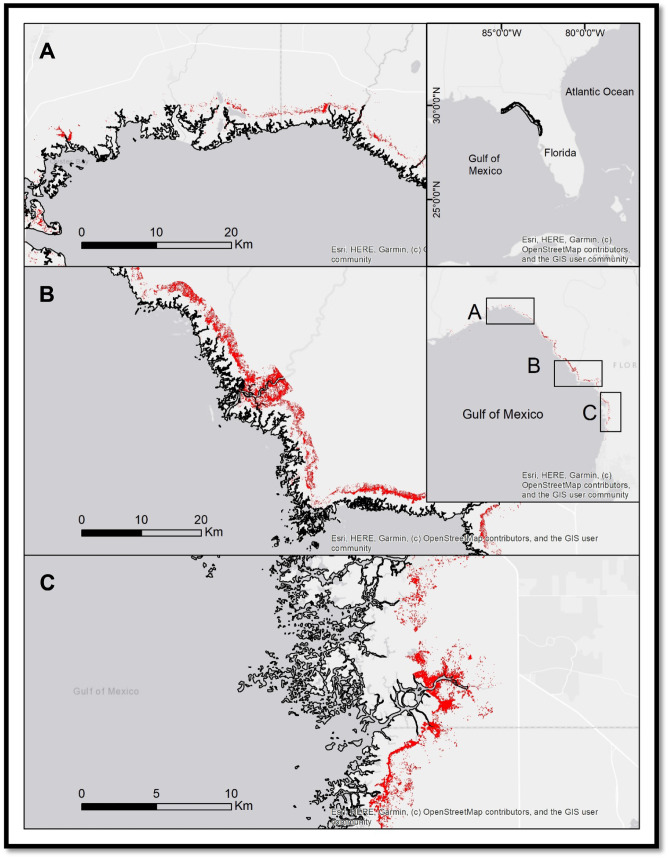
The Big Bend study area of the Florida (USA) Gulf Coast indicating the region of interest (top inset) and areas of coastal forest decline in red (inset maps; background map source: ArcGIS® basemaps)

The coastal areas of northwest Florida along the Gulf of Mexico are low-lying regions of largely undeveloped tidal marsh and hardwood forest. Collectively the region is known as the Big Bend (Fig. 1). As the longest undeveloped stretch of coastline in the contiguous United States, the Big Bend provides insights into the effects of rising seas on critical natural coastal habitats in the absence of anthropogenic mitigation (e.g., seawalls, hardened shoreline, revetments) that may mask or artificially abate inundation (Raabe and Stumpf 2016). Sea-level rise in the Big Bend region is projected to be 22–25% greater than the global average by 2060 (Sweet et al. 2017). Recent research has predicted the loss of coastal forest in the Big Bend region as flooding frequency and salt become more pervasive in the microtidal soil (Williams et al. 1999; Geselbracht et al. 2015; Raabe and Stumpf 2016; Langston et al. 2017). These analyses of historic land cover data and satellite imagery, as well as in situ plots of vegetation composition have observed the pattern of decline and identified the primary driver as sea-level rise and storm surge flooding, but are either limited in spatial extent or outdated relative to recent sea-level rise acceleration.

**Fig. 2:**
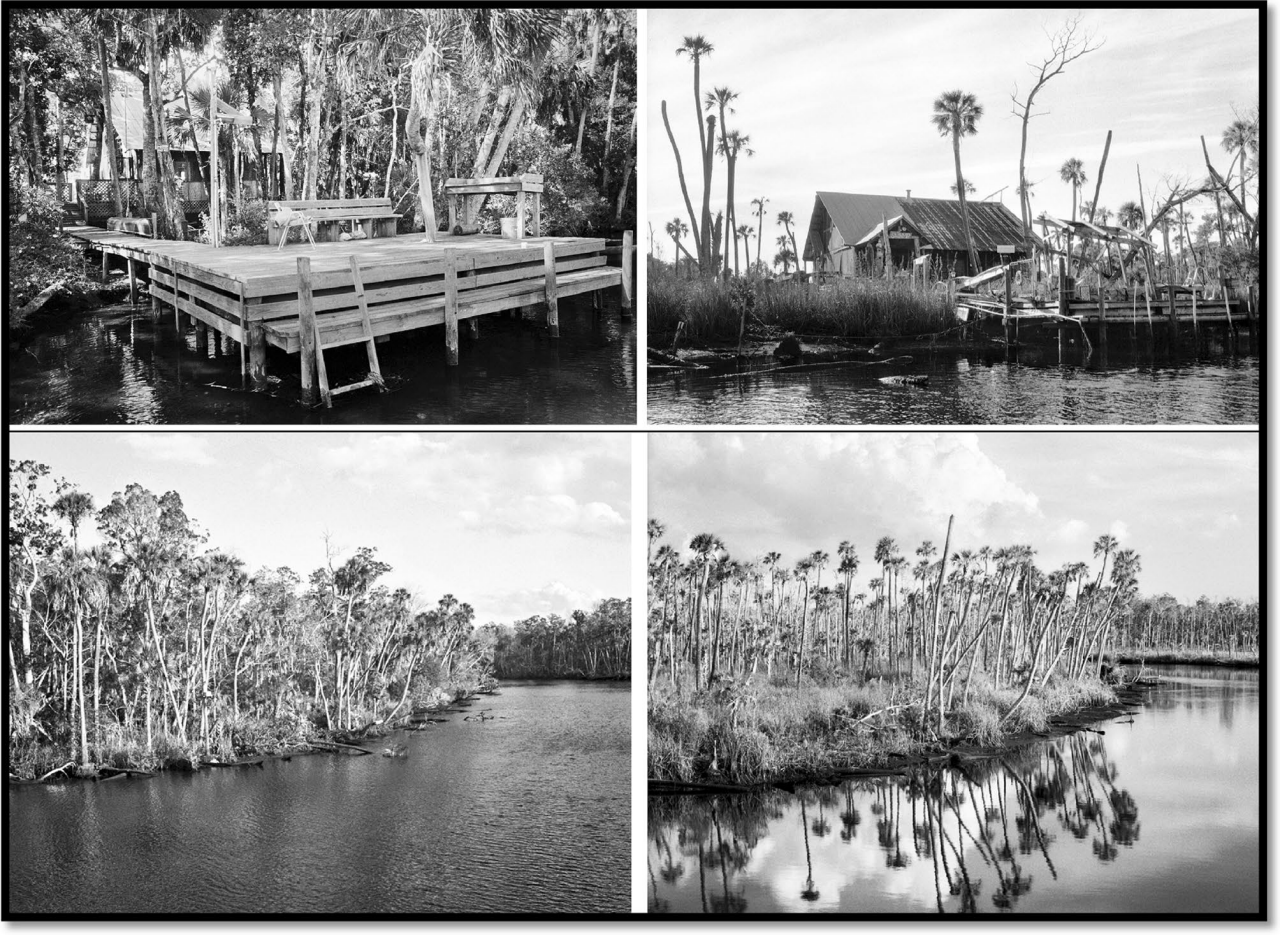
In situ photographs of two sites within the study area taken from approximately the same perspective at different times. (Top panel) Site showing photographs from 1987 (left) and 2017. (Bottom panel) Site showing photographs from 2006 (left) and 2018 (right). These photos reveal a thinning of tree stands and loss of canopy (© Copyright Benjamin Dimmitt)

Further, McCarthy et al. (2018) documented the decline of Sabal palm (*Sabal palmetto*), one the most prevalent tree species in the Big Bend coastal floodplain, within a subset of the region in 2010 (Fig. 1C). In situ photography corroborated the decline and revealed that the loss of forest manifests as a combination of canopy-loss and forest thinning. Our primary goal is to update and expand the analyses with a synoptic quantification of coastal forest decline using satellite and aerial imagery collected between 2003 and 2016 for the entire Big Bend (Fig. 1). Guiding and corroborating the observed patterns are time series of professional landscape photography capturing unprecedented loss of coastal hardwood forest (Fig. 2).

## Materials and Methods

Satellite analyses used cloud-free Landsat 5 and Landsat 8 imagery downloaded from EarthExplorer.usgs.gov. The study area spanned two image footprints (Path/Row 18/39 and 17/40). The before and after images covering the northern footprint were both acquired in the month of October, while the southern images were both acquired in February to avoid seasonal discrepancies in the change calculation for each pair. All images were radiometrically calibrated and converted to top-of-atmosphere reflectance using the ENVI™ software prior to computing Normalized Difference Vegetation Index (NDVI) using the red and near-infrared bands. NDVI is a standard remote sensing index used to identify vegetation from the characteristic low red and high near-infrared reflectance patterns of photosynthesizing material (Zhang et al. 2018). NDVI maps were then restricted to the region of interest as defined by Raabe and Stumpf (2016), before being further restricted to coastal hardwood forest as defined by Geselbracht et al. (2015) using Cooperative Land Cover (CLC v3.2.5) map polygons. By clipping these data to this vegetation dataset, we avoid the need to choose an arbitrary NDVI threshold above which to consider vegetation versus non-vegetation surfaces and avoided areas of silviculture that would bias the results. NDVI values tend to be substantially higher in forested vegetation than grasses, so even if forest loss revealed undergrowth, there should still be a decline in NDVI. NDVI difference was calculated using Raster Calculator in ArcMap v10.5 by subtracting the early map (i.e., 2003) from the later map (i.e., 2015 or 2016). Values greater than or equal to 0 were excluded from further analysis. Artifacts at the edges of the images were clipped out of final products manually. For consistency, we calculated rates based on the more conservative 2003–2016 period rather than parsing the region into northern and southern rates.

A digital elevation model (DEM) was created by downloading and mosaicking 1 arc-second DEM data from The National Map (viewer.nationalmap.gov). To calculate NDVI decline by elevation range, DEM floating point data were converted to integers by rounding up to the nearest integer and extracting those areas that intersect the CLC forest polygons. CLC data excluded silviculture forests, but we additionally restricted NDVI change maps to elevations lower than 1.2 m (4 ft) to further avoid any bias that may be introduced by including inland or upland habitats that may have undergone anthropogenic-driven change through development.

Aerial photographs were downloaded from labins.org for target areas to visually verify the loss of forest as thinned canopies and downed trunks. Google Earth Pro time series imagery was also used to corroborate the loss of forest in these regions as an independent source of verification data. Google Earth Pro collects a multitude of independent imagery sources and collates time series.

To determine the rate of degradation relative to proximity to the Gulf, we created buffer polygons in ArcMap v10.5 from 0 to 2000 m at 500 m intervals from the coastline inland. NDVI decline maps were clipped to each buffered region.

Cedar Key monthly mean higher high water (MHHW) and detrended MSL data were downloaded from tidesandcurrents.noaa.gov for the period 1940–2016 based on the MSL datum. Linear trends were fitted to the monthly MHHW data from the beginning of each decade through 2016. Detrended data had been preprocessed with average seasonal cycle and linear trend removed.

We limited the study period to 2003–2016 to assess chronic drivers of forest decline and avoid incorporating coastal forest degradation caused by the extreme events of Hurricanes Irma (Category 3) and Michael (Category 5) that made landfall along Florida’s Gulf Coast in 2017 and 2018, respectively. During the study period no major hurricanes directly impacted the Big Bend, although Category 1 Hurricane Hermine made landfall in the northern region in September of 2016. Our results, however, indicate limited forest decline in this region relative to the rest of the coast.

## Results

**Fig. 3:**
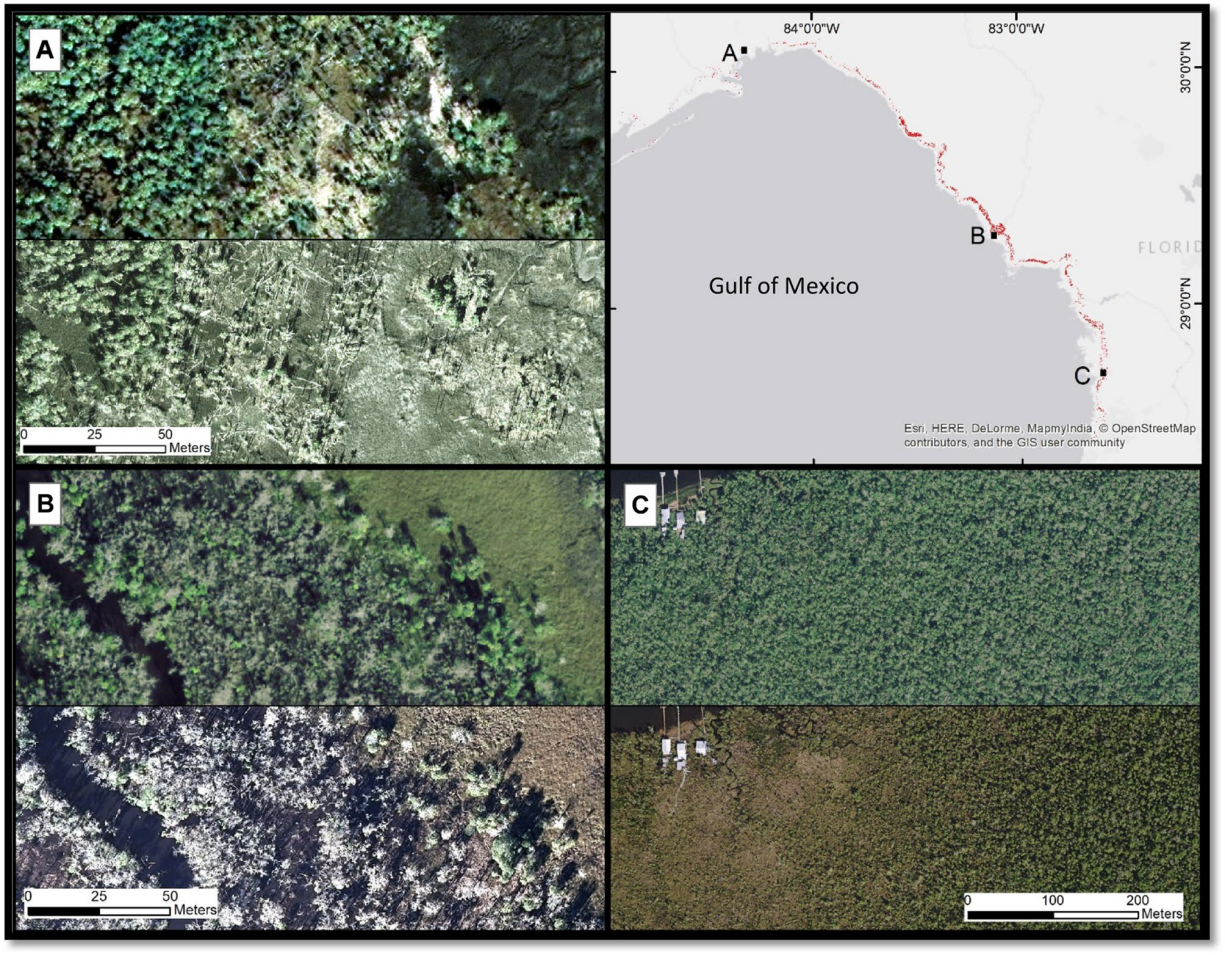
Aerial photography time series pairs of selected areas of coastal forest decline. Forest decline can be visually identified as downed trees or thinned canopies, often amid flooded forest floor. The northern region (**A**; 2006 top, 2016 bottom) exhibited pervasive forest decline along the marsh-forest boundary and along tidal inlets. The central region, especially surrounding the mouth of the Suwannee River, experienced the greatest concentration of decline (**B**; 2008 top, 2016 bottom). Forest within the southern region, especially along the Chassahowitzka River, grows along a slightly shallower elevation gradient and forest stands have been lost widely (**C**; 2006 top, 2017 bottom)

The study area covered 540 km^2^ of coastal forest within 2 km of the coastline to coincide approximately with the region addressed by Raabe and Stumpf (2016) (Fig. 1). We measured the change in coastal hardwood forest over time using the NDVI that we computed using cloud-free Landsat satellite imagery (30 m spatial pixel resolution) from 2003 to 2016 (Fig. 1) (Lunetta et al. 2006; DeVries et al. 2015). Aerial photography (0.3 m resolution) collected over this time frame corroborates the die-off of trees and landward migration of marsh habitat (Fig. 3).

Our results document a decline of 126 km^2^ in forest cover in 13 years (2003–2016), representing 23% of the study area. Elevation data for the study area revealed that forest loss was most prevalent at lower-terrain elevations (i.e., 77 km^2^ < 0.3 m, 38 km^2^ 0.3–0.6 m, 8 km^2^ 0.6–0.9 m, 2 km^2^ 0.9–1.2 m). Forest loss was most rapid along the marsh-forest boundary and in tidal inlets (Figs. 1 and 3).

## Discussion

Raabe and Stumpf (2016) performed a similar evaluation for this region from 1875 to 1995 using historic map data combined with Landsat imagery and found that over this 120-year period, forest cover declined by 148 km^2^. They found that forest decline was not uniform throughout the Big Bend through 1995. Indeed, forests remained healthy along the banks of the Suwannee River. On the other hand, Wahl et al. (2014) found that *S. palmetto* declined rapidly in 2000–2005 in a small area near the center of the Big Bend. This was attributed to the combined effects of sea-level rise and the 1998–2002 La Niña drought (Tully et al. 2019). We filled spatial gaps for which Raabe and Stumpf (2016) could not obtain observations, and we evaluated an additional 21 years of high-quality observations to update the marsh and forest cover assessments using NDVI as a metric of both forest die-off and forest decline (i.e., forest-to-marsh and forest-to-marsh transitional habitat, respectively) as a standard metric for vegetation health (Panday and Ghimire 2012; Lambert et al. 2015). Results indicated a substantial increase in rate of decline from their 1.2 km^2^ year^−1^ to our 9.7 km^2^ year^−1^.

Raabe and Stumpf (2016) concluded that the MHHW level was the primary driver of change at the marsh-forest margin and that MHHW increased at the Cedar Key tide gauge station (8727520) at twice the rate of mean sea level (MSL). We reanalyzed the Cedar Key tide gauge observations through 2016 and found that the rate of MHHW rise has further increased. While the long-term (1940–2016) average rate was 2.56 mm year^−1^, MHHW rose 10.6 mm year^−1^ if averaged over 2000–2016 and 16.5 mm year^−1^ for 2010–2016. This acceleration has resulted in more frequent inundation events observed at the Cedar Key station.

Sea level along this coast exhibits a low frequency signal of 11–14 years with an 18.6-year lunar cycle that may result in periods of rapidly increasing sea level (Stumpf and Haines 1998). Stumpf and Haines (1998) also found short-term (i.e., ~ 5-year) MHHW pulses exceeding 10 mm year^−1^ at Cedar Key during the 1940’s and 1980’s followed by periods of stability or decline. Our analysis indicates a prolonged acceleration beginning around the year 2000. Further, Wahl et al. (2014) evaluated seasonal sea-level cycles for 13 tide gauge stations across the Gulf of Mexico from 1945 through 2011. They found that Cedar Key experienced the highest annual amplitude of all stations, which was 2 cm higher in 2009 than the pre-1990 high. They concluded that these changes have almost doubled the risk of hurricane-induced flooding since the 1990s for this region. Such storm-induced flooding events may also play a substantial role in forest decline, but identifying the relative contribution of acute versus chronic drivers to large-scale forest decline would require additional research utilizing higher temporal resolution data and may still elude attribution if the decline response is lagged. Further, although we avoided the influence of major hurricanes on the study area in our chosen time period, there were six tropical storms that impacted the region during the study period that may have caused some storm surge or wind damage and could not be accounted for here.

The mechanism of sea-level rise-induced flooding of the low-gradient karst landscape is well established, but the rates have increased substantially in the last decade. Although forest die-off has been observed across this region for decades, the death of the forest has accelerated since around 2010 (McCarthy et al. 2018).

The irregular pattern of forest habitat loss that Raabe and Stumpf (2016) documented in the Big Bend region has been replaced by a pattern of pervasive forest habitat loss throughout the region, including the previously resilient forest adjacent to the Suwannee River (Fig. 3B). Decline throughout the region consistently occurs along an advancing marsh-forest boundary and tidal inlets (Fig. 1). The acceleration in the death of the forest is the result of compounded stresses, including acute weather events (i.e., cold snaps and drought periods) and chronic saltwater flooding. This has pushed the ecosystem to a tipping point that both enhances ongoing decline and impedes recovery after the events subsided (Lewis et al. 2016).

Coastal forest along Florida’s Gulf Coast is dying at an unprecedented rate. The rapid die-off of this largely undeveloped forest is resulting in irreversible habitat loss. This likely has impacts on the biodiversity of the region and enhances the physical and socio-economic vulnerability of the few populated areas that dot this coastline (Bradshaw et al. 2009). We expect similar change to occur throughout Florida’s more populated coastal regions where shoreline protections and beach nourishment projects may be delaying the impacts of rising seas (Estevesf and Finkl 1998; Raabe and Stumpf 2016). The types of coastal impacts identified here need to be incorporated into natural resource and urban planning processes to guide planning for adaptation and resiliency throughout the state (Butler et al. 2016).

## Data Availability

Landsat and aerial imagery used are open source and may be downloaded from landsatlook.usgs.gov and labins.org, respectively.
